# CMOS-Compatible Silicon Nanowire Field-Effect Transistor Biosensor: Technology Development toward Commercialization

**DOI:** 10.3390/ma11050785

**Published:** 2018-05-11

**Authors:** Duy Phu Tran, Thuy Thi Thanh Pham, Bernhard Wolfrum, Andreas Offenhäusser, Benjamin Thierry

**Affiliations:** 1 Future Industries Institute and ARC Centre of Excellence for Convergent Nano-Bio Science and Technology, University of South Australia, Mawson Lakes 5095, South Australia, Australia; duy.tran@unisa.edu.au (D.P.T.); thi_thanh_thuy.pham@mymail.unisa.edu.au (T.T.T.P.); 2 Department of Electrical, Electronic and Computer Engineering, Technical University of Munich, 85748 Munich, Germany; bernhard.wolfrum@tum.de; 3 Peter Grünberg Institute, Forschungszentrum JülichGmbH, 52425 Jülich, Germany; a.offenhaeusser@fz-juelich.de

**Keywords:** silicon nanowire, field effect transistor, micro/nanofabrication, CMOS, biosensor, diagnostic, commercialization

## Abstract

Owing to their two-dimensional confinements, silicon nanowires display remarkable optical, magnetic, and electronic properties. Of special interest has been the development of advanced biosensing approaches based on the field effect associated with silicon nanowires (SiNWs). Recent advancements in top-down fabrication technologies have paved the way to large scale production of high density and quality arrays of SiNW field effect transistor (FETs), a critical step towards their integration in real-life biosensing applications. A key requirement toward the fulfilment of SiNW FETs’ promises in the bioanalytical field is their efficient integration within functional devices. Aiming to provide a comprehensive roadmap for the development of SiNW FET based sensing platforms, we critically review and discuss the key design and fabrication aspects relevant to their development and integration within complementary metal-oxide-semiconductor (CMOS) technology.

## 1. Introduction

**Figure 1:**
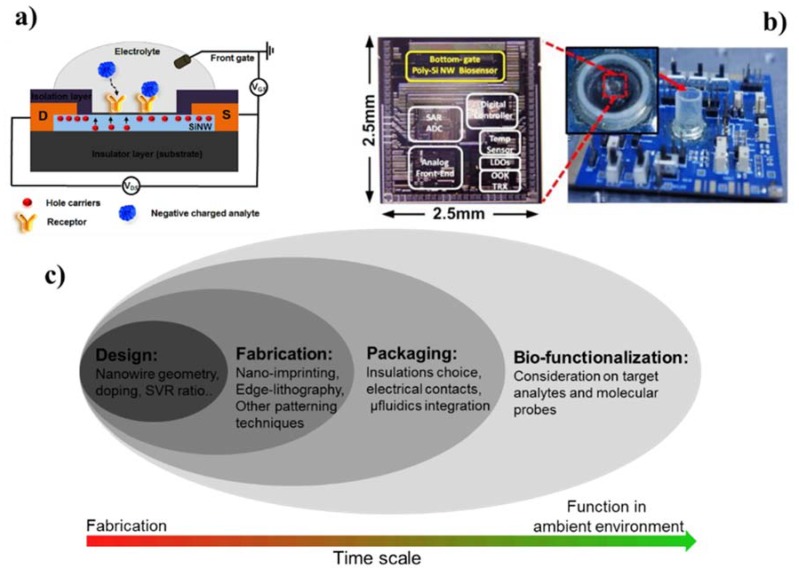
(**a**) Illustration of the biologically active field-effect sensing principle for a *p*-type SiNW FET (cross-sectional view). SiNWs are connected to source (S) and drain (D) electrodes and are biofunctionalized with molecular probes. A front gate electrode is used to modulate the NWs’ conductivity. Specific binding of negatively charged target bioanalytes onto the probes will result in accumulation of positive carriers at the SiNW surface, leading to detectable increases in conductivity. (**b**) Image of an integrated SiNW FET biosensor with implemented poly-SiNW sensing block, microcontroller, temperature sensor, analog-to-digital converter and wireless transceiver. Reprinted with permission from Ref. [21], Copyright (2014), Elsevier. (**c**) Chronological development procedure of a SiNW FET device for molecular sensing application from design, device fabrication, on-chip packaging and molecular probe functionalization.

Biosensor and analytical devices that provide accurate detection and quantification of biological and chemical species have been important to the fields of medical diagnostics, life-science and environmental monitoring. More recently, there has been an increasing demand for development of point-of-care (POC) sensing devices and many promising POC advances based on a number of molecular sensing approaches have been reported for the detection of biomolecules, including electrochemical detection, ELISA, surface plasmon resonance, nanoparticle, quartz crystal microbalance, micro ring resonator, microcantilever, and chemical sensitive field effect transistor [1,2]. However, the inherent shortcomings of these approaches remain a challenge towards their translation in real-life applications, especially those requiring compliance with the Word Health Organization’s ASSURED criteria (Affordable, Sensitive, Specific, User-friendly, Rapid, Equipment-free and Deliverable). Conversely, semiconductor (III–V) nanostructures are one of the most promising nanotechnology building blocks and are actively being integrated in a range of technological applications in nano-electronics [3,4,5], optoelectronics [6,7,8,9], photovoltaics [10,11] and bio/gas sensing [12,13,14,15,16]. This is mostly due to their extraordinary physical properties, for example their ability to be operated as high performance electrical field-effect transistors (FETs) [5,17,18] (e.g., high carrier mobility, high current on/off ratio, and close ideal sub-threshold slope) and excellent photo responsivity [8,9,19]. Among the range of suitable materials and configurations, silicon nanowire field effect transistor (SiNWs-FET) biosensors have attracted significant attention from researchers, funding organizations and for-profit companies due to their compatibility with the existing silicon electronics industry as well as the availability of high-quality and relatively cost-effective substrates. SiNWs-FET biosensors offer high sensitivity, high integration density, high speed sampling, low power consumption, multiplexing, real-time and label-free detection ability [12,13,14,15,20,21]. From a bioanalytical standpoint, the sensing performance of SiNW FETs originate from the concept of biologically active field-effect, where the SiNWs’ conductivities are modulated upon the binding or adsorption of charged (bio)molecules to probes immobilized on their surface [13,22]. For example, the binding of negatively charged molecules on *p*-type SiNWs will drive bulk positive charge carriers towards the nanowires’ surfaces and consequently increase their conductivity, which can be accurately measured (Figure 1a).

A completed SiNW FET biosensor device is a complex sensing system, where each of the functional components (e.g., transducer, readout, functionalization) and detection method are inter-connected and influence each other (Figure 1b). Therefore, it is critical to have a systematic overview on the whole device from fabrication to implementation. This is important not only to successfully fabricate each of the device’s components but also to harmonically integrate and optimize them toward the realization of functional sensors with the level of performance required for a specific application. Although several reviews on SiNW FET fabrication processes have been published [13,23,24,25], a comprehensive and systematic discussion of all key elements of a SiNW FET sensing device is lacking. To this end, we provide here an overview of all the key technological aspects associated with SiNW (bio)analytical sensing devices with an emphasis on state-of-the-art complementary metal-oxide-semiconductor compatible SiNW FETs (Figure 1c). We focus more specifically on key design considerations, fabrication routes and considerations regarding the packaging of such SiNW FETs with functional transducers. We also discuss the sensing performance and reliability of SiNW devices with respect to the whole transducer fabrication aspects, aiming to bridge the gap between theoretical and empirical studies published in the last 10 years. Finally, recent applications of SiNW biochemical sensors in medicine and environmental monitoring are presented along with a discussion about commercialization prospect and remaining challenges.

## 2. CMOS-Compatible Silicon Nanowire Fabrication Techniques

High quality SiNW FETs with various sizes, impurities densities and crystal orientations can be prepared in large quantity using chemical synthesis, for example vapor–liquid–solid synthesis route [26,27,28]. However, synthesized SiNWs have major drawbacks in incorporation to the functional device, namely the lack of control in precise alignment of nanowires (e.g., number, density and fidelity of nanowires), requiring complex transfer of nanowire to the substrate and large devices’ variations in batch-to-batch fabrication. These limitations are roadblocks for large-scale prototype development, device calibration, quality assurance and regulatory approval of any future medical devices based on chemical synthesized SiNW FET. In the other hand, fabrication of SiNW FETs from a wafer using CMOS-compatible top-down approaches typically afford good control over the nanowire dimensions, good fidelity and device homogeneity and readily allow connection with external circuit and readout [29,30,31,32,33,34]. These advantages are resulted from the development of advanced nanofabrication methodologies as well as the availability of high quality silicon on insulator (SOI) wafers. Modern top-down fabrication techniques can be divided into two major groups, vertical and horizontal, depending on the nanowire orientation in relation to its substrate.

### 2.1. Vertical SiNWs

**Figure 2:**
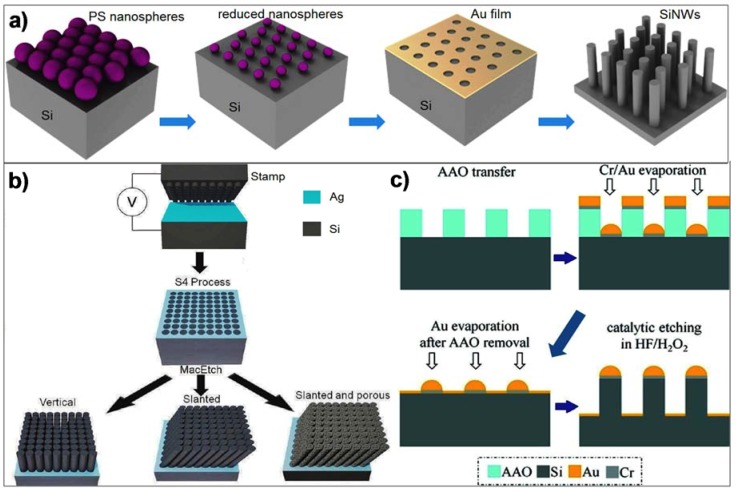
(**a**) Schematic of a typical vertical SiNWs’ fabrication process based on blocking-dots patterning. Polystyrene (PS) nanospheres are deposited on Si wafer, followed by gold (Au) deposition. The resulting Au mesh serves as catalytic mask in the etchant solution (e.g., H_2_O/HF/H_2_O_2_) to define the SiNWs, Reprinted with permission from Ref. [34], Copyright (2011), IOP. (**b**) Vertical SiNWs fabrication by ionic solid-state molding. SiNW pattern is transferred from an Ag_2_S stamp onto an Ag coated Si wafer by applying a potential. Such pattern is used for catalytic wet-etching to form the SiNW, Reprinted with permission from Ref. [39], Copyright (2010), ACS. (**c**) Fabrication of vertical SiNWs by anodized aluminum oxide (AAO) molding. An AAO mold with desired pore diameters is used as a shadow mask for patterning of Au/Cr blocking-dots, which later will be used to define SiNWs through a catalytic wet-etching. Reprinted with permission from Ref. [33], Copyright (2010), ACS.

In the early days of SiNW fabrication, blocking-dots patterning techniques were commonly used as these allow for low-cost patterning of high density, high aspect ratio and random order monocrystalline SiNWs (up to 1 × 10^10^ wires/cm^2^) [34,35]. The nanowires’ diameters, density and spatial distribution can be directly controlled through either a deposited colloidal nanoparticle layer [33,34,35] or lithography assisted nanodot arrays [36,37,38] while the subsequent etching defines the nanowires lengths (Figure 2). Key advantages of this fabrication approach include the mass-production of SiNWs with controlled size and density, highly uniform silicon crystallization with low defects, as well as the fact that they do not require SOI wafers.

### 2.2. Horizontal SiNWs

Top-down nano-patterning techniques usually provide reliable paths to fabricate high quality, high density SiNWs horizontally aligned on planar SOI wafers. Thanks to the maturity of the silicon industry, planar SiNWs can be patterned on large-scale with excellent reliability, quality and spatial resolution. Unlike vertical SiNWs, horizontal SiNW structures can be readily integrated with external electrical circuits, sample delivery platforms as well as with signal processing components. Current fabrication techniques used to fabricate horizontal SiNWs can be categorized into two major sub-groups: nano-imprinting and edge-transfer.

**Figure 3:**
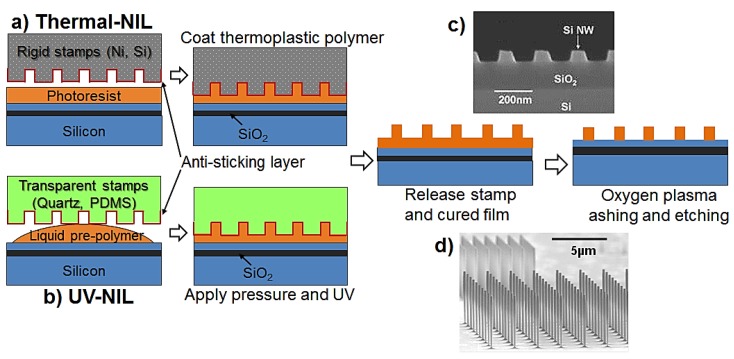
Illustration of SiNWs fabrication processes based on (**a**) T-NIL and (**b**) UV-NIL. Typical horizontal (**c**) and vertical SiNWs (**d**) fabricated by nanoimprinting techniques. Reprinted with permission from Ref. [43], Copyright (2006), AIP and Ref. [7], Copyright (2010), ACS, respectively.

Nano-imprinting technique are bridging the gap between low throughput laboratory-based patterning technology and large-scale production of SiNWs. Nano-imprinting approaches mainly rely on the mechanical transfer of patterned structures from pre-fabricated stamps/masters to a silicon wafer, which is typically followed by a dry/wet-etching step [7,39,40,41]. Depending on the imprinting resist and stamp’ material, diverse SiNW patterns can be structured in the resist by high temperature stamping (e.g., thermal nano-imprinting lithography (T-NIL)) or by UV curing pre-polymers with transparent stamp (UV-NIL) (Figure 3). Using this concept, well-aligned SiNW arrays can be fabricated with high throughput and high fidelity over various structure’s dimensions (~sub-10 nm resolution [42] and up to millimeters in length [40]).

**Figure 4:**
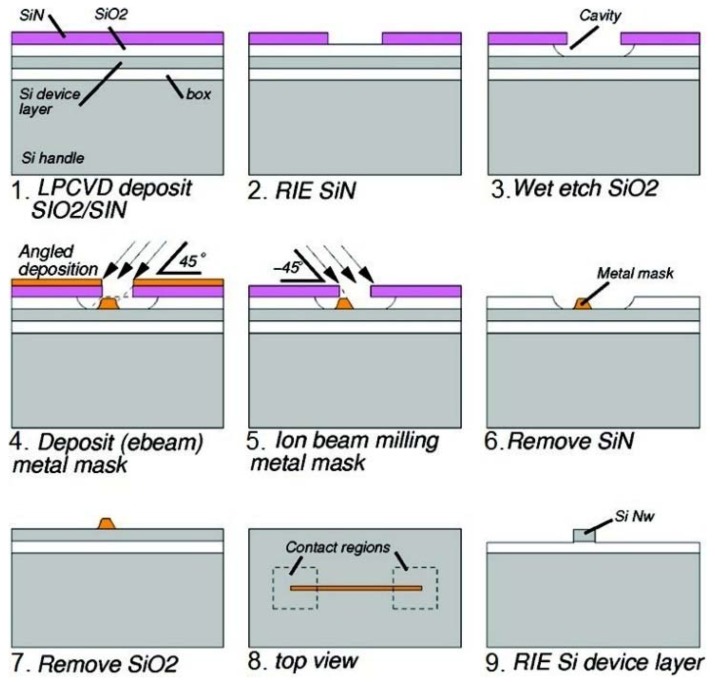
Schematic illustration of SiNW fabrication by the edge-transfer technique: (**1**–**3**) patterning of the nano-cavity by selective wet-etching of SiO_2_ supporting structures with Si_3_N_4_ mask; (**4**–**6**) forming the Cr mask by Ar milling under angle and wet-etching of the supporting structures; and (**7**–**9**) nanowire patterning with dry-etching using defined Cr mask. Reprinted with permission from Ref. [45], Copyright (2009), ACS.

The edge-transfer fabrication approach is a good compromise between performance and cost-effectivity. This approach is designed for patterning SiNWs in various dimensions without the need for advanced nanolithography. Therefore, it is compatible with conventional micro-fabrication facilities. The key principle of this fabrication route relies on creating a mask of deposited materials at the corner/edge of a supported structure. The SiNWs are then defined from the wafer by a typical etching step [44,45] using the pre-defined mask. For example, millimeter SiNWs can be readily fabricated on SOI wafers using an etched mask patterned from the engineered nano-cavity/undercut structures (Figure 4) [44,46,47] or by transforming the vertical thickness of the masking materials into the lateral width of the nanowire patterns [41,48,49,50,51]. Applying this concept, well-ordered SiNWs can be patterned with any desired configurations ranging from sub-10 nm in width [46], millimeters or more in length [45] and with high density [44].

**Figure 5:**
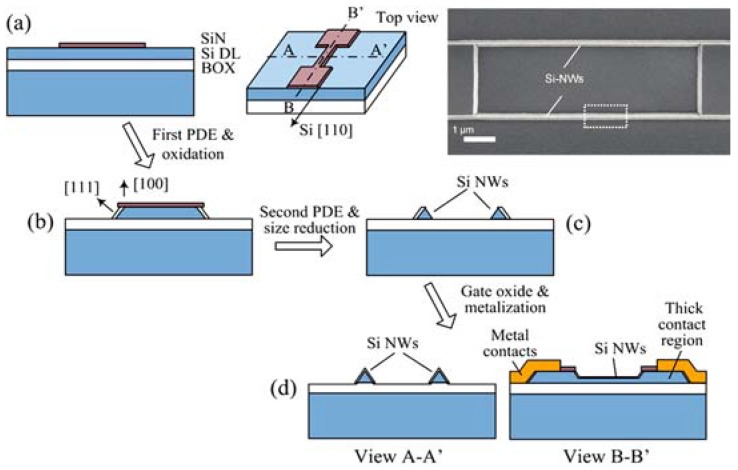
Illustration of SiNWs fabrication using the size reduction method: (**a**) a SiN layer is deposited and then dry-etched to form the mask; (**b**) a plane dependent etching (PDE) is performed to etch out the exposed Si layer followed by oxidation at the [111] side; (**c**) a second PDE and size reduction process are used to realize the SiNWs; and (**d**) the SiNW device is completed through formation of the gate oxide and metallization of the contacts. Inset: SEM image of the resulting triangular SiNWs. Reprinted with permission from Ref. [32]. Copyright (2009), ACS.

In addition to these two established fabrication approaches, several innovative patterning techniques have also been reported. For example, NWs can be fabricated by trimming down from microwires. The patterned Si micro-structures in this method can be narrowed by mean of oxidation trimming [52,53,54] or wet-chemical etching (Figure 5) [32]. Advanced lithography patterning technologies such as electron beam lithography [30,55], dip-pen nanolithography [17], and focused ion beam lithography [56], are useful for fast prototyping owing to their capability to directly pattern the NWs with very high resolution (sub-10 nm structures) without the need of photomasks.

## 3. Integration of Silicon Nanowires into Functional Devices

### 3.1. Forming High-Quality Electrical Contacts

Low-resistance and stable electrical contacts are important for a functional device since its reliability (e.g., trans-conductance, high frequency behaviors) is mainly dominated by the bonding quality between the metal electrodes and the SiNW FETs [18,57,58]. A good electrical contact should possess as small Schottky barrier height as possible to allow for optimal flow of charge carriers between the metal and NWs. For CMOS compatible SiNW FETs, it is common to use Ohmic contacts, which can be achieved by heavily ion-implanting (>10^19^ atoms/cm^3^) the source/drain of SiNW FETs followed by metal deposition and thermal annealing [32,45,59]. Choosing appropriate materials for the electrical contacts is important not only for achieving near ideal sub-threshold slope [58] but also for reducing low-frequency noise and power consumption [60,61]. Various metals and metallic alloys including Al [32,62,63,64], Ti [18,57,65], Pt [66,67,68], Co [69,70], Ni [71,72,73], and Cr [62,64,74] can be vacuum deposited to form good Ohmic contacts with SiNWs. Among these, Al and Al-Si alloys are the most common materials as they can react with oxygen in SiO_2_ residue to form electrical contacts during thermal annealing. However, Al is a highly reactive metal which usually requires the use of a protective metal (e.g., Pt, Au, and Ag) to improve the contact durability. Another approach is to form silicide contacts with SiNWs by sintering the metal on undoped silicon to create metal-silicon alloy. Most frequently metals used in silicide contacts are TiSi_2_ [57,58], CoSi_2_ [75,76] and NiSi. [77,78]. In comparison with Ohmic contacts, silicide contacts are superior in bonding durability, temperature resistance, lower contact resistance (~µΩ), and simpler fabrication process (ion-doping free). The main drawback of this type of contact is the uncontrollable silicon consumption during thermal sintering which may reduce the device reproducibility [77,78].

### 3.2. Dielectric Insulation for Silicon Nanowire Device

In the early days of FET devices, SiO_2_ was mainly used as gate dielectric material due to its large band gap, high resistivity, and compatibility with silicon fabrication industry. With the advent of nano-scaled FET devices (e.g., SiNWs-FET), SiO_2_ is however less compatible for high performance devices—especially in modern nano-electronics and biosensing applications. For instance, ultra-thin SiO_2_ layers can be slowly dissolved in aqueous solution by hydrolysis, particularly in high ionic strength solution [79,80,81]. SiO_2_ gate dielectric is also being limited by a drifting effect of the device’s output current, as well as by gradual incorporation of charged ions from the electrolyte [82,83,84]. These issues can be overcome by using other materials with high dielectric constants such as Si_3_N_4_ (*k* = 7.5), Al_2_O_3_ (*k* = 9), SnO_2_ (*k* = 9.86), HfO_2_ (*k* = 25), and T_2_O_5_ (*k* = 26). With these high-*k* materials, thickness of the gate dielectric can be increased without losing the gating capacitance to the nanowires. Experimental studies on high*-k* material coated SiNW (Al_2_O_3_ [85], T_2_O_5_ [86], HfO_2_ [87,88,89] and stack of HfO_2_/Al_2_O_3_ [83]) showed high stability and almost Nestian pH sensitivity. The utilization of hydrosilylated alkenyl monolayer as organic dielectric insulation is also an interesting approach. However, more investigations need to be done in this approach to improve the device stability [90].

The sensing performance of SiNW FET biosensors is directly dependent on quality of the gate insulation materials. Although growing thermal SiO_2_ on SiNWs is much easier than coating with high*-k* materials, the latter provide significantly improved performance in regard to durability and sensing stability. Among high*-k* materials, Al_2_O_3_ and HfO_2_ are most frequently used for molecular biosensors. However, the implementation of high*-k* materials adds some complexity to the fabrication process as defects can be introduced at the interface between silicon/high*-k* material films [91]. To moderate interface defects and maintain CMOS fabrication compatibility, it is important to select the appropriate high*-k* materials depending on the end-user application. For instance, 10 nm of Al_2_O_3_ coated SiNW is stable up to three months in physiological solutions [81] but the material itself has larger intrinsic defects compared to SiO_2_. This typically results in degradation of the FET device performance later on [91,92]. The deposition of stacks of high*-k* silicate or high*-k* aluminates film is among the most promising approach to solve this issue [93,94]. For instance, Torii et al. demonstrated successful integration of a HfAlO/SiON stack as gate dielectric into a standard CMOS process with relative low leakage (~10^−2^ A/cm^2^), low interface density (2 × 10^11^ eV^−1^ cm^−2^) and relatively high electrical performance.

### 3.3. Sample Delivery: on-Chip Microfluidics Integration

Automated on-chip microfluidic integration is desirable in (bio)analytical applications because it allows the device to operate with small sample amounts (in the orders of nanoliters), multiplex detection capability and fasten response time of the sensor in comparison with static sampling [65,71,95,96,97,98]. Accurate control of the liquid flow is key to the function of microfluidic integrated FET sensing devices. This can be achieved with either passive or actuated microfluidics. In passive microfluidics, liquids are driven into the microfluidics with minimum or no externally powered driving force, for example osmotic pressure, surface chemical gradient, permeable polymer or capillary forces [99,100,101]. In particular, Gao et al. successfully integrated SiNW FET into polydimethylsiloxane microcapillaries using a simple surface treatment with polymer brush and oxygen-plasma bonding [100]. Such SiNW FET integrated microcapillary is appealing for POC sensing applications owing to their relatively simple fabrication, good portability, low dead volume and low power consumption. However, it is complicated to design a passive microfluidic system that can effectively control the flow rate as well as provide amount of sample in demand. Therefore, the majority of SiNW FET sensing devices are currently integrated within actuated microfluidics because they are simpler to fabricate/integrate and offer more control over flow rate, sample amount and are compatible for in-vitro sampling with incubation-washing. Typical driving force of actuated microfluidics are mostly come from electric-field, centrifugal, displacement or magnetic-field pumping mechanisms [102].

Recent research in SiNW FET on-chip microfluidic integration has focused on lowering the detection limit and response time of the sensor. By adding a top-bluff body facing the SiNWs in a straight microfluidics channel, Kim et al. observed a ~124% reduction in reaction time in comparison to a conventional planar microfluidic [103]. Another promising route to ensure efficient transport of the target analytes to the sensing areas is to use a straight microfluidic channel design with simple build-in split flow structures. Such microfluidic design has the advantage of low mechanical influence on the flow velocity while maintaining the hydrodynamic focusing effect [104]. Notably, the electrical performance of SiNWs embedded in microfluidics is significantly influenced by the potential streaming effect of electrolyte’s flow velocity [105]. This could be solved by shielding the sample solution with two integrated reference electrodes placed upstream and downstream of the microfluidics [106]. Finally, similar to other microfluidic biosensing platforms, minimizing non-specific adsorption onto the microfluidic channels is critical. To generate fouling-resistant microfluidics, several surface treatment methods have been successfully utilized including polyethylene glycol based coatings [65,107] or serum albumin [108].

### 3.4. Multichannel Electronic Readouts

(Bio)analytical assays typically require multiplex detection of a panel of target analytes to improve the overall performance. Most current SiNW FET biosensor readout depends on single channel monitoring of conductance/resistance changes with commercial probe stations or semiconductor analyzers [74,109,110,111]. Despite their ultralow-noise and excellent resolution, probe stations require highly trained operators and are bulky, expensive, time consuming to set-up and therefore not compatible for decentralized applications. Customized portable multichannel readouts for SiNW FET biosensor have been developed [21,29,112]. Alternatively, SiNW FET biosensor can also be analyzed with an application specific integrated circuit (ASIC) readout. The ASIC platform readout principle is based on conventional resistance-to-frequency converters, in which the binding of target molecules changes the nanowire’ resistance and hence, change the frequency of the output waveform [113,114]. Using this concept, ASIC readout could allow multiplex measurements in the 1000s-sampling rates per second range, which is useful for applications requiring fast kinetic measurements (e.g., metabolic enzymatic assays).

## 4. Sensing Performance: Finding the Best Compromise among SiNW FETs Fabrication Aspects

### 4.1. Impact of the Nanowire Design in Sensing Performance

More than for any other solid state (bio)sensing technologies (e.g., surface plasmon resonance, electrochemical platforms), the intrinsic performance of SiNW FET biosensor is dramatically influenced by its electronic quality, and therefore critical care must be given to its design and fabrication. Depending on the specific application, there are important fabrication parameters that need to be carefully optimized. It is worth to note that those fabrication parameters are often interrelated and cannot be considered independently.

The cross-sectional dimensions of SiNW FETs are critical toward its (bio)analytical application. Smaller nanowire often provides more sensitivity than larger one, which is partly due to their larger surface to volume (SVR) ratio [41,115,116]. In pH measurement for instance, small SiNWs (50 nm width) exhibited higher conductance changes in response to pH than larger ones (over 150 nm in width) [115]. Similar results have also been reported for biosensing [116] and gas sensing [41]. The size-function dependence of typical n-type SiNWs’ conductance (G) can be described by the concept of SVR ratio:

(1) $$G=\frac{q\text{µ}n}{L}\int_{A}^{}n\;dA$$

where *q* is the electron charge, μ*n* is the electron mobility, *L* is the nanowire length, and *n* is the electron density for each area element A [115].

**Figure 6:**
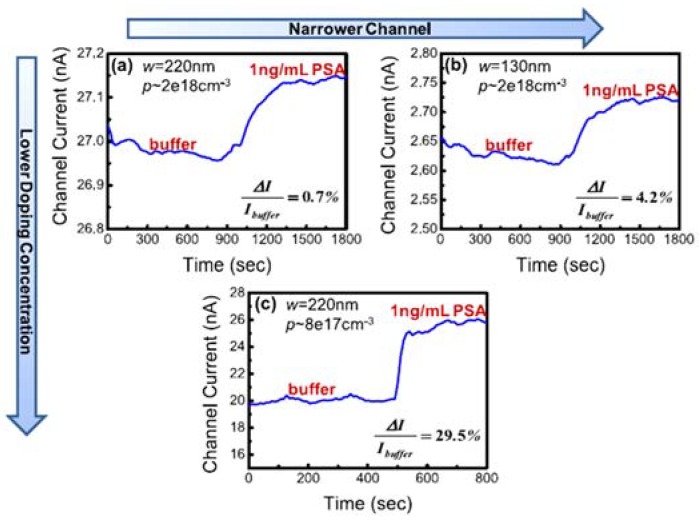
Demonstration of the effects of the SVR and doping concentration on the overall sensitivity for the real-time detection of the prostate serum antigen (PSA) biomarker (1 ng mL^−1^): (**a**) SiNW width = 220 nm and doping concentration ∼2 × 10^18^ cm^−3^; (**b**) SiNW width = 130 nm and doping concentration ∼2 × 10^18^ cm^−3^; and (**c**) SiNW width = 220 nm and doping concentration ∼8 × 10^17^ cm^−3^. Reprinted with permission from Ref. [120], Copyright (2009), IOP.

It is well-known that the doping concentration plays a crucial role in the electrical performance of SiNW FETs. In recent studies, it has been shown that low ion doping concentrations (independent to the ion type) are usually required to achieve high sensitivity SiNW biosensors [116,117,118,119]. For instance, a ~69% increase in sensitivity was experimentally reported when reducing the nanowires’ doping concentration from 10^19^ to 10^17^ atoms cm^−3^ [116]. In comparison to the SVR, doping concentration has a dominant effect in SiNW FET devices sensing performances with up to 400% improvement (Figure 6) [120]. 

**Figure 7:**
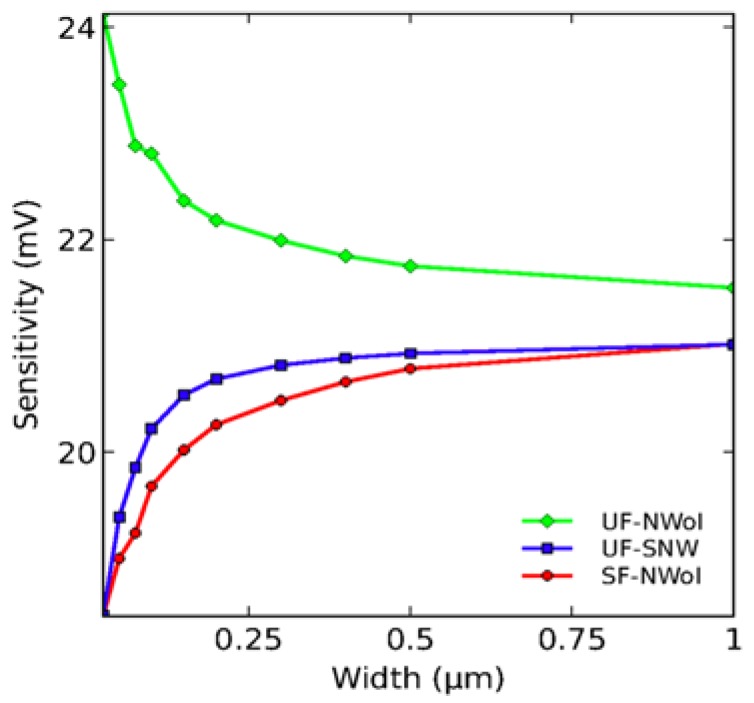
Dependence of the detection sensitivity vs. nanowire widths as a function of SiNW geometry and surface functionalization. While the sensitivity of the non-selective functionalized horizontal SiNWs (UF–NWoI) increases with reducing width, the sensitivities of UF–SNW (non-selective functionalized suspended SiNWs) and SF–NWoI (selective functionalized horizontal SiNWs) decrease with decreasing widths. Reprinted with permission from Ref. [124], Copyright (2014), PNAS.

Beside the strong effects on performance of the SVR and doping level, the NWs overall configuration and geometric arrangement should also be considered. In regard to SiNW cross-sectional shapes (e.g., triangle, rectangular, hexagonal, and circular), it has been suggested the existence of an edge effect which influence the electronics properties [121,122,123]. However, these influences are less significant in comparison with doping levels and SVR parameters. In addition, while it has been predicted that suspended SiNW FETs provide improved sensing performance vs. non-suspended ones [103,119,124], this has been recently disputed owing to the demonstration of a so-called surface-to-Debye-volume ratio effect (Figure 7) [124]. For example, suspended rectangular SiNWs with four convex edges have larger Debye volume than horizontal ones with two convex and two concave corners. Although these results require further experimental validation [103,119], this illustrates the complexity of the structure function relationship for SiNW FET biosensor. A similar scenario can be found in regard to the nanowire lengths. Theoretical calculation predicts shorter SiNWs are more sensitive than longer ones at the same doping concentration [119]. However, longer nanowires are advantageous in regard to the binding kinetic, and present larger sensing areas for binding the target molecules [125,126]. For biosensing applications, the most commonly used lengths are in the range of 2–20 µm.

The response of SiNW FET biosensors is inherently governed by the binding affinity of the target molecules, their concentrations as well as their diffusion kinetic from the bulk liquid at the ultra-low concentrations [14,124,126]. All these factors contribute to the final response time of the biosensor and need to be taken into account in the device design. The total sensing surface area plays an important role in the sensing response time. In this regard, silicon nanoribbons with micrometer widths are more advantageous at ultra-low concentration (sub-pM concentration) than nanowire [126]. For example, nanoribbon biosensors (50 µm length, 50 µm width) require ~1 h to reach the equilibrium state while it takes several days for an equivalent nanoscale structures (10 nm length, 2 µm width) [126]. Although the SVR of nanoribbon sensing FETs is not favorable for maximizing sensitivity, their low intrinsic noise and high electronic stability are usually superior to those of NW FETs.

To compromise between sensitivity and stability, nanowire arrays are commonly used and are considered superior in terms of reliability and reproducibility [95,116,127]. In such arrays, the device sensitivity is averaged from the conductance changes of each individual nanowire [127]. The pitch size plays a role in the sensing performance. For example, a small pitch size array was shown to have reduced sensing performance due to the fringing capacitance and low electrostatic field resulting from the applied back gate voltage [128]. Although the total sensing area of arrays are larger than that of NWs, the device overall sensitivity is still complex and depends on whether the detection is reaction limited or near the end of detection equilibrium.

Sensor’s long-term stability is especially required for applications aimed at live cell-recording and implantable devices. Studies performed with SiNW FET with SiO_2_ dielectric insulation reported an approximately 2–3 weeks of functional stability when recording neuron cells activities [129,130]. By choosing the appropriate dielectric insulation type, it is possible to obtain SiNW FET functionality of up to one year [81]. 

### 4.2. Considerations on Fabrication Route and Process-Selection Frame Work

Top-down vertical SiNWs can be readily fabricated with high aspect ratios, high density, and good uniformity and low cost over large areas. However, the implementation of vertical SiNWs in (bio)sensing device is difficult in regard to integration, packaging and sampling delivery methods. Most SiNW FET biosensors are therefore based on planar nanowire structures. Among the fabrication routes, edge-lithography techniques provide cost-effective fabrication in various structure sizes, diameters, and cross-sectional shapes. However, extra care needs to be taken to reduce the cross-contamination inherently associated with this lengthy fabrication processes. Alternatively, maskless nanolithography approaches (e.g., nanoimprinting, dip-pen nanolithography, electron beam and focus ion beam) are more straightforward and achieve high resolution patterning with low risk of cross-contamination. However, low throughput and high operation cost are the main drawbacks of those techniques. Other techniques, such as nanostencils [131], size reduction [32], and lithographically patterned nanowire by electro deposition [47], are reasonably simple and cost-effective, but are somewhat limited in term of the nanowire’s morphology, density and lateral resolution.

In comparison with monocrystalline SiNWs, polysilicon SiNWs fabricated by edge lithography are desirable when considering the fabrication cost, as their preparation usually does not require expensive SOI wafer and advanced nanofabrication techniques. On the other hand, the fabrication of polysilicon SiNWs requires stringent annealing conditions and their electrical performance (e.g., carrier mobility) and batch-to-batch uniformity are usually not as good as that of monocrystalline nanowires.

In summary, the fabrication of CMOS-compatible SiNW FET biosensor can be presented as a sequential process of selection of the nanowire design, patterning technique, dielectric insulation, metal contact formation, biofunctionalization, microfluidics integration and readout strategy (Table 1).

### 4.3. Molecular Probes and Target Analytes

SiNW FET sensors respond primarily to changes on their surface charge associated to the binding of target (bio)molecules. Thus, attention must be paid to the target molecules’ nature (i.e., surface net-charge and isoelectric point), surface functionalization as well as measurement conditions (pH and ionic strength). Considering the bio-FET detection principle, it is not surprising that most target analytes are highly charged molecules such as proteins, DNA/RNA, virus, or strongly polar molecules. In the case of neutral or weakly charged molecules, adding charges to the molecules is generally required. This can be achieved, for instance by adding reporter molecules [132] or creating bioreaction complex [133,134].

Choosing the right surface receptors is important for the implementation of SiNW FETs as sensing units. Similar to other biosensing technologies, molecular probes ideally should possess high specificity and affinity to their targets as well as remain bioactive upon immobilization. Here, we briefly discussed some key aspects relevant to SiNW FET sensors. Comprehensive reviews are available for antibodies [135,136], enzymes [137], DNA [138] and aptamers [139]. Monoclonal and polyclonal antibodies are commonly used in SiNW FET biosensing, for instance for the detection of protein biomarkers [111,140], virus [97,141,142] and small molecules [143]. Antibodies typically have high binding affinities, excellent specificities to their targets and well-established immobilization protocols are available. The main disadvantage of antibody probes in the context of FET sensing is their relatively large size (~14 nm length), which limits the detection sensitivity in high ionic strength conditions (e.g., Debye length in physiological solution is ~0.8 nm). This issue can be mitigated by the use of small antibody fragments [144,145] or synthetic antibodies [146]. For instance, Roey et al. functionalized the surface of SiNW FETs with fragments of IgG antibodies (ca. 2–3 nm length) and compared the detection performance to identical devices functionalized with whole IgG antibodies. Under physiological conditions (e.g., 1× PBS), the fragment IgG functionalized devices allowed for the sensitive detection of Troponin T protein down to pM concentrations while whole IgG functionalized devices showed only negligible changes in conductivity [144]. Enzymes are mainly used as molecular probes for the detection of non-polar molecules (e.g., glucose and very low molecular weight peptide) as subsequent enzymatic reactions can be used to generate detectable changes in either the surface charge [147,148,149] or to change the solution pH [134]. Nucleic acid-based probes are the third major class of molecular receptors used with SiNW biosensor. This includes single strand DNA (ss-DNA) and PNA [66,98], which are typically used to detect small oligonucleotides. In comparison with antibodies, nucleic acid probes are much smaller, more stable, more cost-effective and provide higher charge density upon binding of the targets.

Finally, the surface functionalization of SiNW FETs is important, as it not only provides the necessary platform to immobilize the probes but also controls ultimately the specific and non-specific binding of the target analytes to their probes. This topic has been covered in recent articles [25,150,151] and will not be discussed in detail here.

## 5. Applications of Silicon Nanowire Biosensor in Biomedical and Environmental Monitoring

### 5.1. Medical Diagnosis Device Development

The exquisite sensitivity and practicality of SiNW FET platforms makes them ideal candidates in a broad range of biodiagnostic and biomedical applications (Table 2). In Table 2, a wide range of typical research topics toward medical applications of SiNW FET are selected and summarized. Among these research topics, development of in vitro diagnostic devices is one of the most prominent application of SiNW FET and has been actively explored in both academic and private research sectors. Recent researches on medical diagnostic SiNW FET focused on the development of integrated platforms compatible with the clinical environment [12,108,152]. For example, Tran et al. described the development of a rapid portable POC device able to detect disseminated tumor cells within tissues and fluids with a limit of detection less than 1 cell/mL within an hour. This study demonstrates the feasibility of developing an ultrasensitive, rapid and portable diagnostic device based on SiNW FET that could be applied within the surgery field [12].

As noted above, SiNW FET platforms are particularly relevant for the detection of genomic biomarkers. Through careful optimization, a limit of detection of the target DNA as low as 0.1 fM could be achieved, as well as high specificity for single-nucleotide polymorphism discrimination [153]. The sensitivity of SiNW FET platforms in fact usually surpass by orders of magnitude the clinically relevant concentration of most biomarkers and non-specific adsorption events, an issue common to all solid-state biosensing technologies, remains a major challenge towards the detection of biomarkers at ultralow concentrations. Signal amplification approaches are a promising way to mitigate this issue. An example of such methodology has been reported based on the rolling circle amplification of the target DNA, which provided an impressive signal to noise ratio of more than 20 for DNA concentrations of 1 fM [148].

Despite numerous reports of successful detection of disease biomarker in buffer solution, direct detection in real samples such as whole blood, serum, and biopsied specimens remains challenging due to the high ionic concentration of biological materials and the large background noise resulting from the non-specific absorption of bio (macro) molecules. To circumvent these issues, Stern et al. developed a pre-enrichment process for sensitive detection of prostate (PSA) and breast cancer (CA 15.3) markers in blood based on a sandwiched approach. [108] To this end, photo cleavable linkers conjugated to capture antibodies are used to first bind the protein markers inside a microfluidics chip. After a washing step, a short UV irradiation step is then performed to release the bound biomarkers towards their subsequent downstream detection using silicon nanoribbon FET biosensor. Concentrations of 2.5 ng mL^−1^ for PSA and 15 U mL^−1^ for CA 15.3 could be detected using this approach. A different concept was proposed by Paltosky et al. [154], where the blood sample was filtered, desalted, and pre-concentrated before performing the measurement. This approach enabled the detection of the target at sub ng mL^−1^ concentrations within 10 min. 

### 5.2. Bio-Pharmaceutical Applications

**Figure 8:**
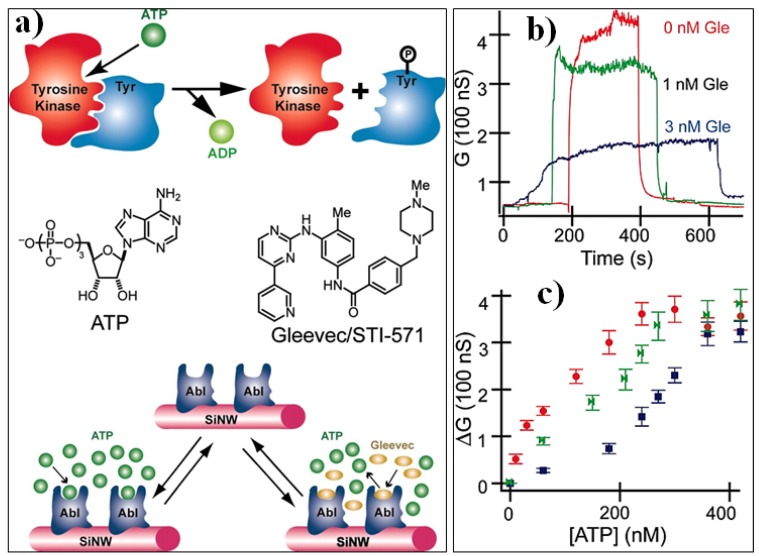
Application of SiNW FET in drug discovery. (**a**) The binding of negatively charged ATP to tyrosine kinase enzyme activates the phosphorylation of tyrosine protein and release ADP. In the presence of uncharged Gleevec molecules, the immobilized tyrosine kinase on the SiNW surfaces is inhibited by the competitive binding of the Gleevec molecules. (**b**) Real-time monitoring of ATP binding to the immobilized tyrosine kinase in the presence of different concentration of Gleevec molecules. The ATP concentration is fixed at 240 nM. (**c**) Conductance change (ΔG) vs. ATP concentration for Abl-functionalized SiNW in the presence of different concentrations of Gleevec: red (0 nM), green (1 nM), and blue (3 nM). Reprinted with permission from Ref. [65]. Copyright (2005), PNAS.

SiNW FET biosensors have also been successfully applied in the bio-pharmaceutical field for the analyses of small molecules activity and screening of new drug molecules. For example, Wang et al. electrically monitored the binding and unbinding of Adenosine Tri-Phosphate (ATP) to protein tyrosine kinases enzyme anchored on the surface of SiNWs by controlling the addition of a drug candidate Gleevec [65]. In this study, the binding of highly negatively charged ATPs to the tyrosine kinase resulted in increasing of the nanowire’s conductance. On the other hand, the competitive binding of uncharged Gleevec molecules to the enzyme prevents the binding of ATP and thus does not impact on the conductance of the nanowires (Figure 8). Using this concept, a minimum inhibition constant of Gleevec was ~2 nM, which is one order of magnitude lower than the general kinetic assay (~25 nM). Sharing the same platform, neuronal transmitters (e.g., dopamine) can also be reliability detected via aptamer modified SiNW arrays [166]. Compared to the conventional electrochemical methods, the sensor shown at least two orders of magnitude sensitivity improvement as well as high specificity. These studies illustrate the excellent potential of SiNW FET in the bio-pharmaceutical field towards the screening and quantitative assessment of small molecules.

SiNW FET proved to be a powerful platform for recording the action potential of neuronal cells with high precision [174,175,176]. High resolution detection of neuronal signals obtained from the SiNW has a significant potential in enabling further understanding of brain functions as well as improved drugs screening for neurological diseases. In a recent study, Kang et al. demonstrated the feasibility of using extended-gate SiNW FET with a custom built-in amplifier to precisely record artificial neuronal signal [175]. Using multi-electrode array as comparative platform, this study provided in-depth understanding of front-end neural signal sensing and design suggestions for further improvement of the platform’s signal-to-noise ratio.

### 5.3. Environmental Monitoring

**Figure 9:**
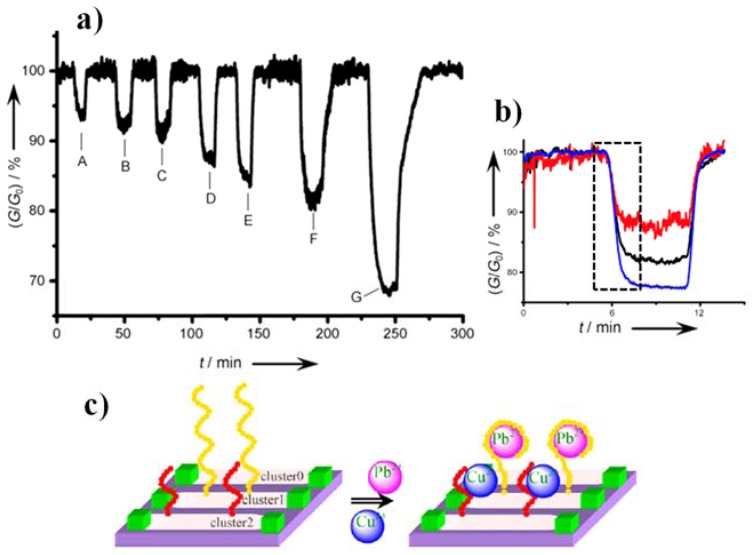
Example of SiNW FET sensor in environmental monitoring. (**a**) Response of an APTES-functionalized p-type SiNW FET when exposed to different concentrations of TNT of and a reference solution: (A) 500 fM; (B) 5 pM; (C) 5 nM; (D) 75 nM; (E) 100 nM; (F) 500 nM; and (G) 5 mM. (**b**) Real-time measurement of TNT (5 μM) in 0.1% DMSO/H_2_O solution in three different devices (black, red and blue curve). The decrease and increase of the nanowire conductance are regulated by adding TNT and reference solution, respectively. The black dash box indicates the time to reach the sensing plateau. Reprinted with permission from Ref. [177], Copyright (2010), Wiley. (**c**) SiNW FETs for the detection of heavy metals through oligopeptide clusters: Cluster 0, aldehyde modified SiNWs; Cluster 1, oligopeptides specific for Pb^2+^; and Cluster 2, oligopeptides specific for Cu^2+^. Reprinted with permission from Ref. [180], Copyright (2009), Elsevier.

Beyond the biomarker and bio pharmaceutical fields, SiNW FET devices have also demonstrated their strong potential to impact on the field of environmental monitoring. The detection of 2,4,6-trinitrotoluene (TNT) is not only important for security reasons but also from the environmental pollution standpoint. Trace concentration of TNT in solution could be detected in real-time at sub-femtomolar level (<10^−15^) using SiNWs modified with monolayer of 3-aminopropyltriethoxysilane (APTES) (Figure 9a,b) [177]. It is hypothesized that TNT molecules form strong charged complexes with the APTES layer which efficiently impact on the SiNWs conductivity. This approach provided ultra-high sensitivity (as low as 10^−2^ ppt in air), short time response (ca. minutes) and long-term stability (over 4 h) for the detection of TNT from real air samples without pre-purification. This illustrates the specificity and feasibility of SiNW FET for application in security and environmental pollutant screening. Similar studies have also reported on the detection of volatile organic compounds such as alkane vapors, alcohols, ethers, benzenes and ketones [178,179].

Airborne biological transfection is critical not only for public health but also for security concern. Real-time monitoring of airborne biological contaminants using SiNW FET incorporated in wireless modules was also reported [20]. Aerosol H3N2 virus samples are first transferred to solution using the bioaerosol-to-hydrosol air sampling techniques. The aqueous solution containing the virus samples are then delivered to the SiNW FET devices for sensing measurement. The conductance changes resulting from the binding of the target virus is then transmitted remotely to a computer or cell phone for monitoring. Approximately 20–30% increases in the sensor response was observed as the virus concentration increases by 10-fold. Moreover, the selective and rapid responses of SiNW FET were also demonstrated for the detection of H1N1 virus in house dust allergens. Further development of this approach is particularly promising for the monitoring of airborne pollution as well as that of biological threats.

Due to their extreme toxicity, the detection of contaminant metal ions (e.g., Hg^2+^, Cd^2+^, Pb^2+^, and Cu^2+^) is critical in water monitoring, food processing as well as medicine. By functionalizing SiNW FET with 3-mercaptopropyltriethoxysilane (MPTES), Lou et al. demonstrated the highly sensitive detection of Hg^2+^ and Cd^2+^ in aqueous solution at concentrations of 10^−7^ and 10^−4^ M, respectively [181]. The selective detection of Pb^2+^ and Cu^2+^ could also be achieved using metal an ion selective oligonucleotide modified SiNWs-FETs device [180]. Limit of detections as low as 1 nM for Pb^2+^ and 10 nM for Cu^2+^ were obtained. Although the specificity of such sensor was not specified, this study illustrated the promising nature of SiNWs-FET sensing platforms for water pollution monitoring and clinical toxicology applications.

## 6. Challenges and Opportunities of Silicon Nanowire Biosensor

### 6.1. Early-Stage Development Platforms

Owing to the aforementioned excellent characteristics of SiNW FET biosensing platforms, a number of initiatives in both the academic and for-profit sectors are aimed at developing commercial technologies. However, these SiNW FET biosensing platforms are still at the research and development phase and yet to received FDA or CE/ISO approval for routine application.

Pioneers in SiNW FET biosensor development, Charles Lieber and his team at Vista Therapeutics, Inc. (Santa Fe, US), a spin-off company from Harvard University (US), first introduced SiNW FET biosensor prototypes for various application in cancer diagnostic, virus and biological toxin detection. The company provides a complete solution from sample processing, on-chip platform, readout instrument as well as data processing [182]. As presented in Table 3, most of the companies active in the field are spin-offs from University research groups. However, global corporations IMEC and Roche have recently joined the race for nanowire FET biosensor development with their newly founded companies miDIAGNOSTICS and BiomedX, respectively.

### 6.2. Challenges and Needs for Commercialization of Silicon Nanowire Biosensor

Despite significant technological advances, the commercialization of CMOS compatible SiNW FET biosensor technology is still in the early phase. Similar to other solid-state sensing technologies, significant challenges remain to be solved and further improvements are required to fulfill the intrinsic promises of SiNW FET sensing platforms and allow for their routine implementation. Thanks to the progress in microfabrication technology, large-scale patterning and CMOS integration of SiNW FET is no longer an issue. However, practical challenges associated with high manufacturing cost still need to be resolved. In addition, quality assurance in large scale fabrication of such device is also required to insure the required device homogeneity necessary for regulatory accreditation. Another significant challenge is the measurement paradigm. In the conventional DC measurement in SiNW FET platform, the total conductance changes are measured against charged target molecules concentration in low ionic solution. Development of either new sample processing or new measurement approaches are needed toward fast, reliable and reproducible diagnostic devices. High performance multiplexed conductivity measurement of SiNW FET biosensor also remains an issue in real-life implementation, and the development of bench-top multiple channel readout with ultra-low-noise and high resolution is required.

## 7. Conclusions and Outlook

The development of SiNW FET molecular biosensing platforms has been critically discussed from their design and fabrication to functionalization perspectives. Fabrication of high performance SiNW FET biosensor requires the fine balancing of many key factors that influence their sensitivity, stability and reliability. It is therefore difficult to define the ideal characteristics of SiNW FETs and rather specific designs should be considered depending on the targeted applications. As a general rule, SiNW devices with low numbers of nanowires, low doping concentrations and short nanowire channel lengths are expected to display maximal sensitivity but may be limited by lack of stability. Increasing the SiNW surface to volume ratio is also expected to improve the device sensitivity but the effect of the cross-sectional dimension on reproducibility and reliability remains unclear. Further theoretical and experimental studies are required to fully elucidate the structure–function relationship of SiNW FET sensors. Choosing optimum fabrication route, packaging strategy, sample delivery and readout methods requires a careful balancing between benefits and drawbacks of each approaches. These technological keynotes must be taken into account in view of the specific applications. Despite several challenges that still need to be addressed, SiNW biosensing platforms are rapidly being developed in the private sectors, yielding the prospect of an imminent paradigm shift in medical diagnostic and environmental monitoring.

## Figures and Tables

**Table 1:** Fabrication process selection frame work of SiNW FET biosensor according to application requirements.

Step	Selection	Possible Options and Their Characteristics	
		Option A	Option B
1	Silicon crystallization type	Monocrystalline. Mostly available on SOI wafer, expensive, best electronics properties and uniformity	Polycrystalline. Require deposition and thermal annealing. Cheap, relative good electronic properties, uniformity depend on deposition and annealing process
2	Nanowire designs	Low-concentration doping (<10^17^ cm^−3^) yields higher detection sensitivity	High-concentration doping (>10^17^ cm^−3^) yields lower detection sensitivity
		Smaller NWs yield higher SVR and hence more sensitivity to charge detection	Larger cross-sectional NWs result in lower SVR and lower detection sensitivity
		Single NW: Theoretically better LODs but prone to large detection signal variation and batch to batch variation	NW arrays: Better reliability but lower limit of detections (LODs)
3	Top-down fabrication routes	Horizontal NWs: High density, good control over quality and uniformity, compatible to packaging and integration but require advanced facility and have high fabrication cost.	Vertical silicon NWs: High aspect ratio, high density, good uniformity and low cost but challenging in regard to integration, packaging and sample delivery
3’	Horizontal SiNW fabrication approaches	Edge transfer lithography: Compatible with standard microfabrication facilities, low cost but lengthy process and high risk of contamination	Nanoimprinting and advanced nanolithography: Require advanced facilities but have low contamination risk and good fabrication throughput and high cost
4	Dielectric insulation	Thermal SiO_2_: Compatible with standard fabrication process, low intrinsic defects but unstable in long-term measurement in liquid	High-*k* materials: Additional fabrication complexity and prone to interface defects but excellent durability and stability in long-term measurement
5	Electrical contact formation	Ohmic contacts: Standard process, requires ion-implantation at the contact areas and sensitive to temperature	Silicide contacts: Require stringent control in metal deposition and annealing steps but provide excellent electrical contacts and no requirement for ion-implantation
6	Microfluidics integration	Active microfluidics: Simple fabrication, operation and integration with SiNW FETs but require external power source or pump	Passive microfluidics: Compatible with POC applications, low cost, low or zero energy consumption but careful design and fabrication required to control sample flow and integration with NW FETs
8	Readout	Multi channels: Multiplexed sensing capabilities. Mainly custom built based on the specific specification of the SiNW FETs. Require compromise between resolution/functionality and cost	Single channel: Commercial read out available offering high resolution and universal functionality but bulky, expensive and not compatible for multiplex and POC sensing

**Table 2:** Bioanalytical applications of SiNW FET platform.

Sensing Type	Fabrication Approach	Probe	Analyte	LOD	Ref.
**Gas, VOCs**	Nano imprinting, bottom-up synthesis	Bare, Silane	NO_2_, VOCs	20 ppb	[155,156,157,158]
**Ions**	Advanced lithography, nanoimprinting	Bare, chelated protein, aptamer	H^+^, Ca^2+^, K^+^	1 µM	[29,159,160,161]
**Small molecules**	Size reduction, advanced lithography, edge transfer	Glucose oxidase, benzylphosphonic acid-4-boronic acid pinnacol ester, phenylboronic acid, aptamer	Glucose, dopamine	1 fM	[162,163,164,165,166]
**Genetic materials**	Size reduction, advanced lithography	DNA, PNA, aptamer	DNA, cDNA, RNA	0.1 fM	[148,153,167,168]
**Protein markers**	Nanoimprinting, Size reduction, advanced lithography	Antibody	Protein	30 aM	[12,108,169]
**Pathogen**	Advanced lithography, bottom-up synthesis	Antibody	Bacteria toxin, virus materials, virus particles	1 fM	[97,141,142,143,170]
**Cell, tissue, organ**	Nanoimprinting, bottom-up synthesis	Fibronectin, silane	Cardiac cells, neural cell/tissue, heart	N/A	[112,171,172,173,174]

**Table 3:** Early commercial SiNW FET platforms and targeted applications.

Company	Product	Detection Targets	Format	Ref.
Vista therapeutics, Inc. (US)	NanoBioSensor™	Cancer markers, viruses, biological toxin, DNA/RNA	Sample collection/Prep Kits; NanoCards; Readout devices	[182]
QuantuMDx, (UK)	Q-POC™	Cancer marker, bacteria, virus, DNA/RNA	Sample cartridge; readout device	[183]
Nanosens, B.V. (The Netherlands)	Nanowire in various materials, nanowire on-chip	Under development	Wafer-scale nanowires; chip-unit	[184]
Inanobio, LLC. (US)	Fully depleted exponentially coupled SiNW FET on-chip	Under development. Co-develop with medical devices company	Wafer-scale nanowires; chip-unit	[185]
Tracense (Israel)	TESS explosives trace detector	Gas sensing platforms for explosive, chemical and biological warfare agents detection	Detection unit: nanowire chip; readout device	[186]
Avails Medical, Inc. (US)	Under-development	Drug monitoring in saliva	Under-development	[187]
NanoIVD, Inc. (US)	Under-development	Genetic mutation detection	Test cartridge	[188]
miDIAGNOSTICS (Belgium)	Under-development	Proteins, nucleic acids, and small molecules	Under-development	[189]
BiomedX	Under-development	electrolytes, proteins, and blood gases	Under-development	[190]
